# Metronidazole-Induced Encephalopathy in a Patient with End-Stage Liver Disease

**DOI:** 10.1155/2012/209258

**Published:** 2012-12-17

**Authors:** John P. Knorr, Imran Javed, Neha Sahni, Ceylan Z. Cankurtaran, Jorge A. Ortiz

**Affiliations:** ^1^Department of Pharmacy, Einstein Medical Center Philadelphia, 5501 Old York Road, Philadelphia, PA 19141, USA; ^2^Department of Transplant Surgery, Einstein Medical Center Philadelphia, Philadelphia, PA 19141, USA; ^3^Department of Hepatology, Einstein Medical Center Philadelphia, Philadelphia, PA 19141, USA; ^4^Department of Radiology, Einstein Medical Center Philadelphia, Philadelphia, PA 19141, USA

## Abstract

*Purpose*. Metronidazole-induced encephalopathy (MIE) has been rarely reported. We report a case in a patient with end-stage liver disease (ESLD). *Summary*. A 63-year-old male with ESLD secondary to hepatitis C virus presented with progressively worsening fatigue, slurred speech, aphasia, vomiting, and left-sided facial droop after completing a 2-week course of metronidazole for recurrent *Clostridium difficile*-associated diarrhea. He completed a previous course of metronidazole 3 weeks prior to presentation. He is on the liver transplant waiting list and has known hepatic encephalopathy. MRI revealed hyperintense T2 signals involving the bilateral dentate nuclei, inferior colliculi and splenium of the corpus callosum, and increased diffusion restriction at the splenium of the corpus callosum. His neurological function improved over the next several days. He underwent liver transplantation 6 days after admission. A follow-up MRI 6 weeks after presentation revealed resolution of abnormalities; however, paresthesias persisted 6 months after MIE diagnosis. *Conclusion*. An ESLD patient with hepatic encephalopathy developed MIE after a relatively short course of metronidazole. Metronidazole has been shown to accumulate in patients with ESLD. Increased awareness for neurotoxicity when using metronidazole in ESLD patients is warranted, especially in those with potentially confounding hepatic encephalopathy.

## 1. Introduction

Metronidazole-induced encephalopathy has been identified as an adverse effect of prolonged metronidazole use; however, there have only been limited cases reported to date [1–12]. Metronidazole is commonly prescribed for *Clostridium difficile* infections, but may also be used in the management of anaerobic bacterial infections, protozoal infections, *Helicobacter pylori* gastritis, noninfectious colitis, and hepatic encephalopathy. While generally well tolerated, the most common adverse drug reactions observed in patients undergoing treatment with metronidazole include nausea, dysgeusia, anorexia, and abdominal cramping [13]. Neurotoxicity has been rarely reported, with features ranging from headache, incoordination, and ataxia to convulsive seizures, optic neuropathy, peripheral neuropathy, and encephalopathy. While the exact incidence and mechanism of metronidazole-induced encephalopathy (MIE) is not known, most cases in the literature have occurred after long-term, high-cumulative dose treatment with metronidazole. We report a case of MIE that occurred in a patient with end-stage liver disease (ESLD) after a relatively short-treatment course for *Clostridium difficile* colitis.

## 2. Case Report

A 63-year-old African American male with ESLD secondary to hepatitis C virus and hepatocellular carcinoma presented to the emergency department with vomiting, general fatigue, slurring of speech, aphasia, and a left-sided facial droop, which was reported as progressively worsening over the past 3 days. He had cirrhosis which was complicated by hepatic encephalopathy and portal hypertension including bleeding esophageal varices and ascites. Prior to admission his medications included nadolol 40 mg daily lisinopril 10 mg daily ferrous sulfate 325 mg 3 times daily, omeprazole 20 mg twice daily, tamsulosin 0.4 mg daily, fish oil 1000 mg daily, and rifaximin 550 mg twice daily. At admission, he was on day 14 of metronidazole 500 mg 3 times daily, which he was prescribed for recurrent *Clostridium difficile*-associated diarrhea (CDAD). His first case of CDAD was diagnosed 5 weeks earlier; he had since completed an initial 14-day course of metronidazole 500 mg 3 times daily without event. During the initial case of CDAD, the patient was started on rifaximin to replace lactulose for maintenance of hepatic encephalopathy prevention.

In the emergency department, the patient received an additional dose of metronidazole 500 mg orally; however, it was not continued upon admission since it was determined that he had completed his course of treatment for recurrent CDAD. The patient was very drowsy but easily arousable to alert and oriented × 3; however, on neurological exam he was found to have dysarthria, diplopia, left-sided facial droop, a positive Romberg's sign, horizontal nystagmus, and bilaterallypositive finger to nose test. Asterixis was absent. The patient's liver function tests were unchanged from baseline, and his ammonia level was within normal limits. His pertinent lab values were as follows: creatinine 1.5 mg/dL, INR 1.5, total bilirubin 1.2 mg/dL, AST 56 IU/L, ALT 41 IU/L, albumin 1.9 g/L, ammonia 28 mmol/L, MELD score 16, Childs-Pugh score 10 (Class C). 

**Figure 1:**
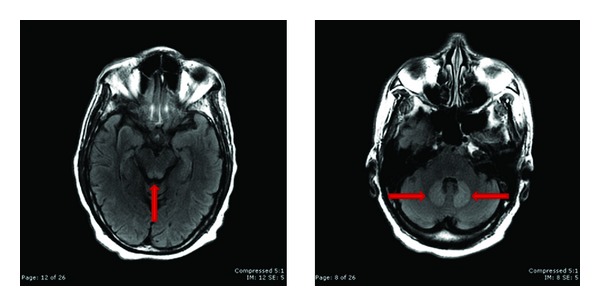
FLAIR sequences at the level of the cerebellum and the brainstem demonstrate hyperintense T2 signal in the bilateral dentate nuclei and inferior colliculi. No diffusion signal abnormality was identified (not shown).

**Figure 2:**
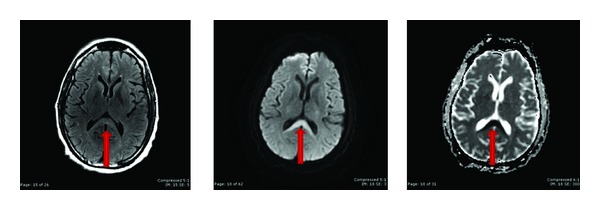
FLAIR and DWI sequences with corresponding ADC map at the level of corpus callosum splenium show hyperintense T2 signal and diffusion restriction compatible with cytotoxic edema.

The initial presentation was suggestive of a cerebrovascular accident with likely cerebellar involvement; however, the CT scan on admission was negative for acute hemorrhage. An MRI showed hyperintense T2 signal involving the bilateral dentate nuclei, the inferior colliculi, and the splenium of the corpus callosum (Figures 1 and 2). There was increased diffusion restriction at the splenium of the corpus callosum only (Figure 2). These findings were consistent with a toxic/metabolic process and with the given history were suggestive of metronidazole-induced encephalopathy.

Six days following admission, the patient underwent deceased donor liver transplantation. His posttransplant course was generally unremarkable, and his neurological function improved over the first posttransplant week. A follow-up MRI after 6 weeks revealed interval resolution of abnormal restricted diffusion in splenium of corpus callosum as well as resolution of abnormal signals involving bilateral dentate nuclei, inferior colliculi, and splenium of corpus callosum (not pictured). While the patient's mental status changes have resolved entirely, he continued to report persistent tingling of his hands and fingers 6 months after diagnosis of MIE.

## 3. Discussion

Metronidazole is the drug of choice for mild-to-moderate, uncomplicated *Clostridium difficile* infections [14]. It is relatively safe; however, associated-neurotoxicity has been reported [1–12]. The clinical presentation of metronidazole-induced encephalopathy in these cases varied. However, most cases presented with ataxia and dysarthria. Other signs and symptoms included mental status changes, peripheral neuropathy, weakness, vertigo, nausea, vomiting, sensory losses, visual disturbances, or seizures. The onset of symptoms has been reported after periods of therapy exceeding 2–4 weeks, with cumulative doses ranging from 21 to 182 grams; however a few reports were in shorter treatment courses. In most cases, symptoms and MRI findings resolve after withdrawal of metronidazole; however, persistent paresthesias have been documented. Our patient experienced complete reversal of his central encephalopathy symptoms and reversal of MRI findings, 10 days and 6 weeks after discontinuation of metronidazole, respectively. Paresthesias persisted 6 months after initial presentation with MIE.

While the exact mechanism of metronidazole-induced encephalopathy is not entirely understood, pharmacokinetic studies have demonstrated that metronidazole crosses the blood-brain barrier and achieves therapeutic concentrations in cerebrospinal fluid [13, 15]. MRI findings most often demonstrate bilateral involvement of axonal swelling with increased water content, inferring a toxic-metabolic process [12]. Another suggested mechanism includes the possibility of vascular spasms that may produce mild reversible localized ischemia [12]. Modulation of the gamma-aminobutyric acid (GABA) receptors within the cerebellar and vestibular systems has also been proposed as possible mechanisms for metronidazole-induced encephalopathy [16].

Ahmed et al. first described the MRI findings of metronidazole-induced encephalopathy as lesions in the dentate nuclei of the cerebellum, corpus callosum, basal ganglia, and frontal and subcortical white matter [12]. MRI findings of bilateral involvement of the dentate nuclei are a very characteristic feature of metronidazole-induced encephalopathy and should be used to distinguish between MIE and other possible causes of encephalopathy [12, 17]. In comparison, classic MRI abnormalities associated with hepatic encephalopathy include high signal intensity in the globus pallidum on T1-weighted images [18]. 

Metronidazole and its metabolites are primarily excreted in the urine; however, up to 60% of metronidazole first undergoes metabolism via hepatic oxidation, and to a lesser extent by glucuronidation [13, 15]. While dose reduction is not required in patients with mild-moderate hepatic dysfunction, accumulation of metronidazole and its hepatic metabolites become apparent in patients with severe liver dysfunction. Loft et al. conducted a pharmacokinetic study in patients with alcoholic cirrhosis and grade 2–4 hepatic encephalopathy who were treated with single doses of metronidazole [19]. They demonstrated that the clearance of metronidazole was reduced to 35% and that the half-life was extended to 280% in cirrhotic patients, when compared to healthy controls. In the four patients who continued therapy for hepatic encephalopathy, there were no signs of accumulation of the parent compound; however, the primary metabolite accumulated in 2 of the 4 patients after 6 days. It should be noted that due to its primary metabolism through oxidation, there are no clinically relevant drug interactions that would increase the risk of metronidazole toxicity; however, the manufacturer warns that “simultaneous use of drugs that decrease microsomal liver enzyme activity may prolong half-life and decrease plasma clearance of metronidazole” [20]. 

In addition to this case, there are two other reports of metronidazole-induced encephalopathy in patients with liver disease. Cheong et al. reported a case of new-onset encephalopathy in a 57-year-old male with alcoholic cirrhosis [4]. He presented with dysarthria, ataxia, and confusion 25 days after initiation of metronidazole 500 mg three times daily for hepatic encephalopathy (cumulative 30 grams). The patient resumed normal consciousness after 2 days, and extremity weakness resolved after 2 weeks. Additionally, Galvez et al. reported a case of worsening encephalopathy in a 60-year-old male with chronic liver disease secondary to hepatitis C [9]. Their patient had mild chorea and ataxia at baseline, but presented with worsening symptoms and new-onset dysarthria and myoclonus after only a few days of a metronidazole dose increase. Both patients had MRI findings which were consistent with previous reported cases of metronidazole-induced encephalopathy. 

When evaluating a patient for possible MIE with existing hepatic encephalopathy, it would be prudent to consider known factors that can precipitate or worsen encephalopathy such as sepsis; gastrointestinal bleed; constipation, bowel obstruction, or ileus; dehydration or uremia; nonadherence to lactulose; hepatocellular carcinoma; or acute on chronic liver injury [21]. Concomitant medications should be reviewed since CNS depressants may worsen or mask symptoms of hepatic encephalopathy and worsening liver function could augment the toxicity of hepatically cleared drugs. Additionally, patients who underwent transjugular intrahepatic portosystemic shunt (TIPS) are at considerable risk for hepatic encephalopathy within the first few days after-procedure. The patient presented in this case had resolving *Clostridium difficile*-associated diarrhea, but was not septic or dehydrated. Liver function tests and hepatocellular carcinoma were stable at presentation.

In further analyzing this patient's case with the Naranjo et al. adverse drug reaction (ADR) probability scale, the calculated score corresponded with metronidazole being a “*possible*” cause of this ADR [22]. We feel that the probability score was confounded by the fact that this patient was known to have hepatic encephalopathy; however, as clinical and MRI findings were consistent with previous reports of metronidazole-induced encephalopathy, we feel strongly that metronidazole was the cause of his new-onset neurologic dysfunction. 

## 4. Conclusion

Neurotoxicity is a rare adverse reaction of metronidazole, which is associated with long-term, high-cumulative dose therapy; however, this reaction has also been reported with shorter courses. Patients with severe hepatic dysfunction are at an increased risk of accumulation and may be at an increased risk of metronidazole-induced encephalopathy, even with short-course therapy. We would encourage using the lowest doses and shortest courses possible when using metronidazole in patients with severe liver disease. When using metronidazole in patients with ESLD patients with hepatic encephalopathy, vigilant monitoring for neurologic changes is warranted. Careful interpretation of medical history and MRI should aid the clinician in differentiating metronidazole-induced encephalopathy from worsening hepatic encephalopathy.
